# Navigating Diagnostic Overlaps: A Case Report of Paranoid Schizophrenia, Borderline Personality Disorder, and Hidden PTSD in a Patient with an Orphanage Background

**DOI:** 10.1192/j.eurpsy.2025.1963

**Published:** 2025-08-26

**Authors:** F. S. Pricope

**Affiliations:** 1 Drug Addiction Department, Cluj County Emergency Clinical Hospital, Cluj-Napoca , Romania

## Abstract

**Introduction:**

Diagnosing psychiatric disorders in individuals raised in orphanages is challenging due to symptom overlap. Trauma from institutional life can mimic symptoms of both Post-Traumatic Stress Disorder (PTSD) and paranoid schizophrenia (Hermenau et al. *J Trauma Stress* 2011; 24: 513-516). For example, PTSD symptoms like intrusive memories may resemble schizophrenia’s delusions when trauma affects threat perception. Additionally, PTSD-related attachment issues can exacerbate paranoia (Patel et al. *J Trauma Dissociation* 2016; 17: 123-136). Accurate diagnosis requires careful assessment of trauma history and symptom differentiation (Robinaugh et al. *Depress Anxiety* 2011; 28: 305-311).

**Objectives:**

Challenges of symptoms overlapping in schizophrenia, borderline personality disorder, and PTSD.

Effects of orphanage background and early trauma on psychiatric symptoms.

Diagnostic methods and evolving treatment plans.

**Methods:**

Patient ZN, a 37-year-old female with paranoid schizophrenia and borderline personality disorder (BPD), has had 11 admissions over six years at our clinic. Despite treatment with DSM-5 criteria, PANSS, and BEST scales, using antipsychotics, mood stabilizers, and benzodiazepines, there was no significant improvement. This year, her hospitalizations increased, particularly after developing a strong attachment and maternal feelings toward a doctor who treated her three times consecutively. PTSD, relevant due to her orphanage background and initially unassessed, was later identified through screening. Data were recorded through clinical notes, informed consent was obtained, and the case study’s single-case design limits generalizability.

**Results:**

**Conclusions**

In conclusion, diagnosing PTSD, paranoid schizophrenia and BPD in orphanage-raised adults is challenging due to trauma, overlapping symptoms, and disrupted attachment. This case highlights the complexities of diagnosis and treatment, emphasizing the need for a comprehensive and adaptable approach.

**Disclosure of Interest:**

None Declared

